# Clinical value of quantitative analysis of Sonazoid-contrast enhanced ultrasound combined with shear wave elastography in discriminating and diagnosing breast tumor characteristics

**DOI:** 10.3389/fonc.2025.1485671

**Published:** 2025-05-20

**Authors:** Gang Wang, Yingmei Ouyang, LiTao Ruan

**Affiliations:** ^1^ Ultrasound Imaging Department, The First Affiliated Hospital of Xi’an Jiaotong University, Xi’an, Shaanxi, China; ^2^ Department of Ultrasound Medicine, The Shaoyang Central Hospital of Hunan Province, Shaoyang, Hunan, China

**Keywords:** breast tumors, characteristic, quantitative analysis of contrast-enhanced ultrasound (CEUS), shear wave elastography (SWE), diagnostic efficacy

## Abstract

**Objective:**

This research intends to probe the clinical value of combining Sonazoid-contrast-enhanced ultrasound (S-CEUS) quantitative analysis with shear wave elastography (SWE) for discriminating and diagnosing the nature of breast tumors.

**Methods:**

A total of 108 breast tumor patients (comprising 120 breast lesions) who were classified as category 4 breast tumor cases and underwent routine ultrasound examinations (June 2022-June 2023) were selected for this study. S-CEUS and SWE examinations were conducted on these breast lesions. The morphological characteristics of S-CEUS were assessed, including morphology (regular, irregular), boundary (clear, unclear), and internal enhancement (no enhancement, homogeneous enhancement, heterogeneous enhancement), along with dynamic enhancement features. Additionally, the maximum Young’s modulus (Emax) from SWE examinations was recorded, and the results were compared to the gold standard in pathology. The diagnostic efficacy of S-CEUS quantitative analysis, SWE alone, or their combined assessment in determining the nature of breast tumors was evaluated.

**Results:**

Among the cohort of 108 patients, a total of 120 category 4 breast lesions were analyzed, revealing 68 cases (56.67%) of pathologically confirmed malignant breast tumors and 52 cases (43.33%) of benign breast tumors. Malignant breast tumors exhibited irregular morphology, unclear boundaries, heterogeneous internal enhancement, high enhancement levels, increased enhancement ranges, perfusion defects, and predominantly washout-type time-signal intensity curve patterns. These characteristics were significantly more prevalent in malignant tumors compared to benign tumors (p<0.05). Furthermore, quantitative assessment denoted that malignant breast tumors showcased higher CEUS quantitative scores than benign tumors (p<0.05). The Emax for malignant breast tumors was (91.36 ± 24.15) kPa, which was considerably higher than that for benign breast tumors [(49.86 ± 20.31) kPa] (*t*=9.981, p<0.05). Receiver operating characteristic (ROC) curve analysis demonstrated favorable diagnostic performance in evaluating the nature of breast tumors using S-CEUS quantitative analysis (AUC: 0.845) or SWE alone (AUC: 0.789). Particularly noteworthy was the optimal diagnostic efficacy achieved through the combined assessment of S-CEUS quantitative analysis and SWE (AUC: 0.916), yielding sensitivities and specificities of 95.59% and 80.77%, respectively.

**Conclusion:**

Both S-CEUS quantitative analysis and SWE are valuable tools for the evaluation of benign and malignant characteristics in breast tumors. Particularly, their combined application demonstrates superior diagnostic efficacy.

## 1 Introduction

Breast tumors in the female population predominantly present as malignancies (1). According to the latest cancer burden data (2), the global incidence of breast cancer reached 2.26 million new cases in 2020, with 685,000 deaths, thereby surpassing lung cancer to become the leading malignancy worldwide. Furthermore, within the context of population growth and aging, it is projected that by 2040, new cases of breast cancer will exceed 3 million, with 1 million fatalities anticipated. In light of this, enhancing diagnostic strategies for individuals at high risk of breast cancer, specifically those with breast tumor diseases, is of paramount importance. Early differentiation between benign and malignant breast tumors aids in formulating crucial guidance for treatment approaches. International guidelines (3–5) endorse mammography as the preferred imaging modality for breast cancer detection. However, in China, the majority of women exhibit smaller breast sizes and dense breast tissue, rendering the sensitivity of such examinations relatively low. As advocated by the “Chinese Guidelines for Breast Cancer Screening in Women” (6), routine ultrasound examination offers advantages such as simplicity of operation, non-invasiveness, and absence of radiation, making it the primary imaging method for breast tumor diseases. This approach involves observing characteristics such as lesion location, shape, orientation, margin, echo type, presence of calcifications, blood flow, and presence of enlarged lymph nodes. Guided by Breast Imaging Reporting and Data System (BI-RADS 5th edition), it partially aids in discerning the benign or malignant nature of breast tumors. Nevertheless, the malignant risk for breast lesions determined as BI-RADS category 4 ranges widely from 3% to 94%, making precise diagnosis challenging. Therefore, the utility of routine ultrasound examination for ascertaining breast tumor characteristics has certain limitations (7).

Due to the distinct blood perfusion characteristics between benign and malignant breast masses, real-time acquisition of the entire vascular phase performance through contrast-enhanced ultrasound (CEUS) examination contributes to enhancing the differentiation of breast tumor properties. Furthermore, with the development of second-generation ultrasound contrast agents (UCAs), Sonazoid, a contrast agent composed primarily of perfluoro-n-butane, has been introduced to the domestic market. Unlike SonoVue, which is widely utilized domestically, after bolus injection, Sonazoid can traverse the pulmonary capillary bed to the left heart cavity and then circulate throughout the body. Additionally, the microbubble surface can effectively generate backscatter of emitted ultrasound waves, thereby strengthening the contrast between intraluminal blood and surrounding tissues. As a result, improved ultrasound imaging effects are attainable, holding promise for further advancement in differentiating the nature of breast tumors (8, 9). Moreover, hardness, as a fundamental characteristic of tissues, can reflect the deformability of tissues under stress. Typically, malignant mammary tissues exhibit greater hardness than benign mammary tissues. Hence, real-time shear wave elastography (SWE) examination, allowing for quantitative determination of Young’s modulus values of tumor lesions, also aids in discerning the nature of breast tumors (10, 11). However, up to the present, quantitative standards for Sonazoid-contrast-enhanced ultrasound (S-CEUS) are lacking, and there is limited research concerning its combination with SWE for confirming the benign or malignant nature of breast tumors. This paper integrates the clinical morphological features and dynamic enhancement characteristics of breast tumor patients at the Central Hospital of Shaoyang employing the Chinese preliminary 5-point scoring system for quantitative assessment in conjunction with SWE. The objective is to explore the practical clinical applicability of this combined inspection approach.

Although BI-RADS based conventional ultrasound is widely used in breast cancer screening, its diagnostic accuracy for BI-RADS category 4 lesions remains limited due to overlapping features between benign and malignant tumors. CEUS and SWE each offer improvements but also have inherent shortcomings when used independently. CEUS can misinterpret benign inflammatory lesions with high perfusion as malignant, while SWE may be confounded by soft malignant histologies such as mucinous carcinoma or by variability in ROI selection. S-CEUS provides real-time, dynamic visualization of tumor vascularization and perfusion heterogeneity, which are critical indicators of malignancy. SWE offers quantitative stiffness measurements (e.g., Emax), reflecting tissue mechanical properties affected by tumor infiltration and fibrosis. These two modalities probe different but complementary tumor characteristics—vascular and biomechanical.

This study is based on the hypothesis that the combination of S-CEUS and SWE offers superior diagnostic accuracy for differentiating benign and malignant breast tumors compared to either modality alone. By leveraging the complementary strengths of S-CEUS in evaluating vascular perfusion and SWE in quantifying tissue stiffness, this integrated approach is expected to reduce false positives and false negatives in BI-RADS category 4 lesions. Ultimately, this could help minimize unnecessary biopsies, enable more precise treatment planning, and improve early detection of malignancies, thereby positively influencing patient outcomes.

## 2 Materials and methods

### 2.1 General data

A total of 108 patients (comprising 120 breast lesions) diagnosed with category 4 breast tumors according to the 5th edition of the Breast Imaging Reporting and Data System (BI-RADS), who underwent routine ultrasound examinations at the Central Hospital of Shaoyang from June 2022 to June 2023, were selected for inclusion in this study. Inclusion criteria were as follows: 1) meeting the indications for Sonazoid-contrast-enhanced ultrasound (S-CEUS) and shear wave elastography (SWE) examination; 2) all patients were female and without breast implants; 3) informed consent from patients and their family members for participation in the study. Exclusion criteria included: 1) allergies to the Sonazoid contrast agent; 2) pregnancy or lactation; 3) concurrent presence of other malignant tumors; 4) severe hepatic or renal dysfunction; 5) suboptimal image quality. The age range of the participants was 20 to 79 years, with a mean age of (50.16 ± 9.27) years. The diameter of the breast lesions ranged from 0.72 to 5.10 cm, with a mean diameter of (2.08 ± 0.63) cm. Ethical approval for this research was obtained from the Ethics Committee of the Central Hospital of Shaoyang (No. KY-2022-002-05).

### 2.2 Examination methods

Following the guidelines provided by the “Expert Consensus on Quality Control of Breast Disease Ultrasound Examination (2019 edition)” (12) and the “Expert Consensus on Breast Imaging Examination and Diagnostic Standards” (13), patients were placed in a supine position with hands raised behind the head, exposing both breasts and axillae. A Logiq E9 color Doppler ultrasonic diagnosis apparatus (General Electric, USA) equipped with a 6–15 MHz linear array probe was employed for conventional ultrasound scanning. The assessment included measurements of lesion size, evaluation of lesion location, shape, orientation, margin, echo type, presence of calcifications, blood flow status, and presence of enlarged lymph nodes. In the 2D imaging mode, the largest long axial section of the lesion was selected, and then the SWE mode was activated. The probe was positioned gently and kept stable, ensuring that the SWE sampling frame covered the entire lesion and surrounding normal glandular or adipose tissues. Patients were instructed to hold their breath, and the “continuous excitation” mode was adopted. After a stable image was achieved within 3 to 5 seconds, the color within the sampling frame became stably filled, displaying clear abnormal hardness of the lesion without motion or compression artifacts. Once a stable SWE image was obtained, frame freezing and image capture were performed. Tissue hardness colors were set, with high hardness indicated by red and low hardness by blue. A region of interest (ROI) with a diameter of 1 mm was placed at the hardest point of the lesion to measure the maximum Young’s modulus value (Emax), and three repeated measurements were averaged. After the completion of the SWE examination, the diagnostic device was switched to the contrast-enhanced ultrasound (CEUS) mode. Patients were instructed to remain in a supine position, and a bolus injection of 4.8 mL of Sonazoid contrast agent (approval number: H20180046; manufacturer: GE HEALTHCARE AS) along with 5 mL of normal saline was administered through the superficial vein of the elbow. Dynamic images were stored in real-time to record the entire process while maintaining the probe in the optimal plane (clear image and complete section). After an hour of observation in a resting position, the probe was adjusted to visualize different sections until the contrast agent disappeared. Dynamic images were acquired and recorded, and the morphological characteristics [primarily focusing on shape (regular, irregular), margin (clear, unclear), internal enhancement (no enhancement, homogeneous enhancement, heterogeneous enhancement)] and dynamic enhancement features [enhancement levels (low, iso-enhancing, high), enhancement range (equivalent, increased), presence of perfusion defects (present, absent), time-signal intensity curve (TIC) patterns (persistent, plateau, washout)] of S-CEUS were observed, with the Chinese preliminary 5-point scoring system for CEUS harnessed for quantification.

Quantitative criteria (14): 1 point (lesions with no enhancement but clear boundaries), 2 points (lesions enhanced synchronously with surrounding tissues, unclear enhancement image boundaries), 3 points (lesions enhanced earlier than surrounding tissues, homogeneous or heterogeneous, enhancement range equivalent to 2D imaging, clear boundaries, regular morphology), 4 points (lesions enhanced much earlier than surrounding tissues, typically heterogeneous enhancement, enhancement range larger than 2D imaging, boundaries remaining clear, with or without perfusion defects, no crab claw-like enhancement, irregular morphology), and 5 points (heterogeneous enhancement, enhancement range larger than 2D imaging, relatively earlier enhancement, presence or absence of perfusion defects, typical crab claw-like enhancement, unclear boundaries, irregular morphology). Upon completion, two senior radiologists (with 11 and 16 years of experience, respectively) performed a double-blind assessment of the ultrasonograms and compared the assessment results. In cases where there were discrepancies in the evaluation results, consensus was reached through consultation with a third-party imaging expert.

### 2.3 Observation of indicators

The morphological features of S-CEUS were observed, and quantitative analysis was conducted based on the Chinese preliminary 5-point scoring system for CEUS. The Emax value obtained from SWE examinations was evaluated. These quantitative assessments were then compared with the gold standard in pathology to evaluate the diagnostic efficacy of S-CEUS quantitative analysis, SWE alone, or their combined assessment for discerning the nature of breast tumors. Pathological standards: Breast tumor tissue specimens were harvested and sent to the pathology department of the Central Hospital of Shaoyang for histological analysis, referencing the 5th edition of the World Health Organization (WHO) classification of breast tumors (15). Malignant breast tumors included invasive ductal carcinoma, invasive lobular carcinoma, intraductal carcinoma *in situ*, medullary carcinoma, mucinous carcinoma, and mixed carcinoma, among others. Benign breast tumors encompassed hyperplasia of mammary glands, fibroadenoma, adenosis, intraductal papilloma, inflammatory granuloma, phylloides tumor, and others.

### 2.4 Statistical analysis

Statistical analysis was conducted with the assistance of SPSS22.0 software (Ins., Chicago, IL, USA, Copyright: SPSS Inc). Enumeration data were displayed as “%” and analyzed through the χ^2^ test. Normally distributed measurement data were represented as “ ± s” and analyzed via the t-test. Receiver operating characteristic (ROC) curves were taken to evaluate the diagnostic performance of S-CEUS quantitative analysis, SWE alone, or their combined evaluation for confirming the nature of breast tumors. When p<0.05, variances were considered statistically significant.

## 3 Results

### 3.1 Statistics of pathological outcomes

A total of 120 category 4 breast lesions from 108 breast tumor patients were subjected to pathological confirmation, unraveling that 68 lesions (56.67%) were diagnosed as malignant breast tumors. Specifically, among the malignant cases, there were 53 cases of invasive ductal carcinoma, 3 cases of invasive lobular carcinoma, 6 cases of intraductal carcinoma *in situ*, 2 cases of medullary carcinoma, 1 case of mucinous carcinoma, and 3 cases of mixed carcinoma. The remaining 52 lesions (43.33%) were identified as benign breast tumors, comprising 14 cases of hyperplasia of mammary glands, 22 cases of fibroadenoma, 3 cases of adenosis, 6 cases of intraductal papilloma, 5 cases of inflammatory granuloma, and 2 cases of phylloides tumor.

### 3.2 Morphological features, arterial enhancement characteristics, and quantitative scoring analysis of S-CEUS

Malignant breast tumors displayed characteristics such as irregular morphology, indistinct boundaries, heterogeneous internal enhancement, high enhancement levels, enlarged enhancement range, presence of perfusion defects, and predominantly washout-type TIC patterns. These phenomena were more prevalent in malignant tumors as opposed to benign tumors, with the difference holding statistical significance (p<0.05). Additionally, quantitative assessment based on the Chinese preliminary 5-point scoring system for CEUS suggested that the CEUS quantitative scores for malignant breast tumors were higher than those for benign breast tumors, with the disparity containing statistical significance (p<0.05), as displayed in **Table 1**.

**Table 1:** Morphological features, arterial enhancement characteristics, and quantitative scoring analysis of S-CEUS [n (%)].

Morphological features		Malignant breast tumors (n=68)	Benign breast tumors (n=52)	*t/χ^2^*	*P*
Morphology	Regular	20	42	31.123	<0.001
	Irregular	48	10		
Boundary	Clear	23	41	23.999	<0.001
	Unclear	45	11		
Internal enhancement	No enhancement	0	6	14.919	0.001
	Homogeneous enhancement	18	37		
	Heterogeneous enhancement	50	9		
Enhancement level	Low	6	15	14.873	0.001
	Iso-enhancing	20	22		
	High	42	15		
Enhancement range	Equivalent	29	34	6.109	0.013
	Increased	39	18		
Perfusion defect	Presence	42	13	16.043	<0.001
	Absence	26	39		
TIC pattern	Persistent	6	29	37.956	<0.001
	Plateau	27	18		
	Washout	35	5		
CEUS quantitative scoring		3.77 ± 0.76	2.42 ± 0.76	9.642	<0.001

### 3.3 Statistical analysis of Emax obtained from SWE examination

The Emax value for malignant breast tumors was (91.36 ± 24.15) kPa, which was remarkably higher compared to benign breast tumors with a value of (49.86 ± 20.31) kPa, and the variance held statistical significance (*t*=9.981, p<0.05).

### 3.4 Analysis of typical images and pathological results

**Figure 1:**
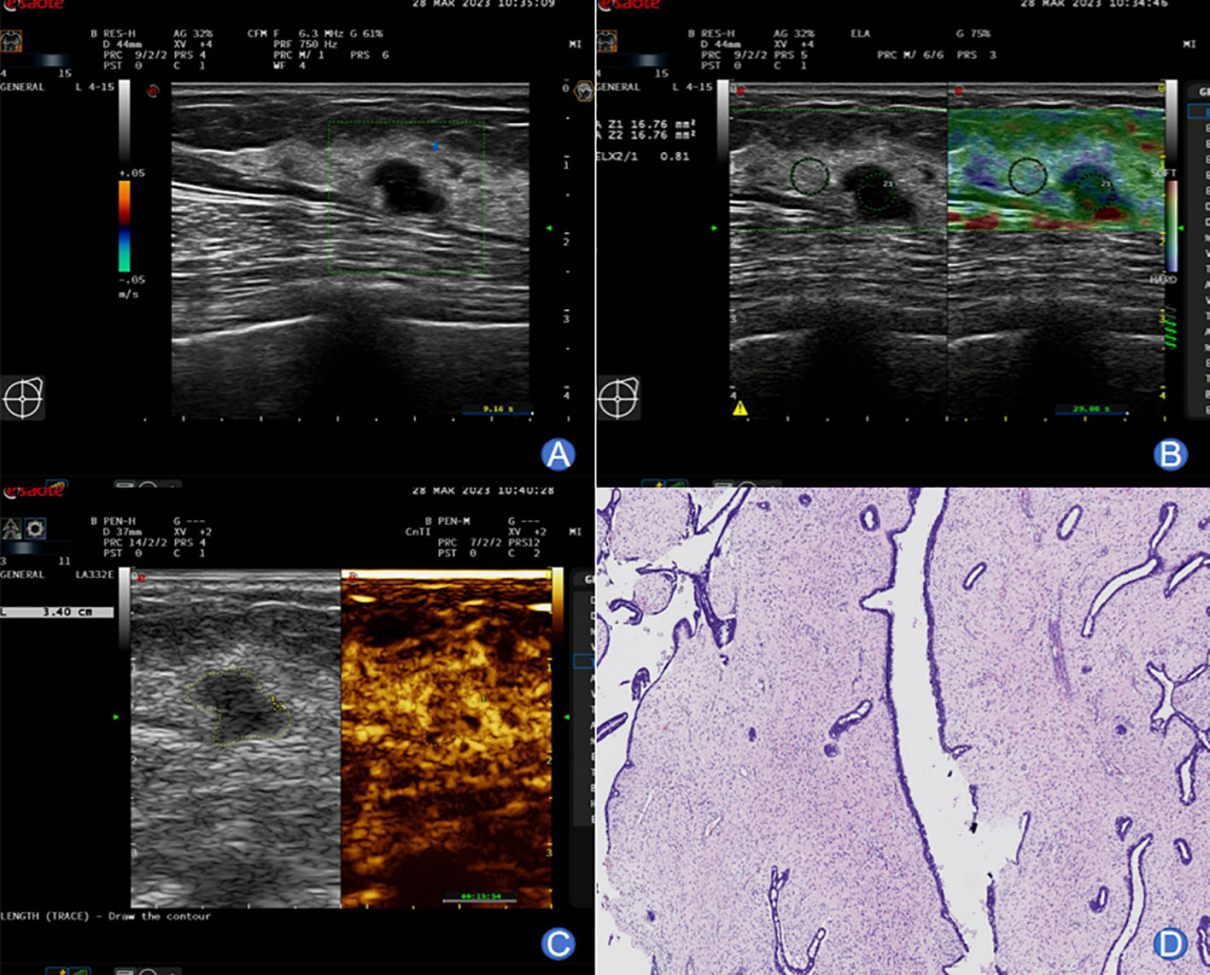
A 49-year-old female with typical images of benign breast tumor. **(A)** Conventional ultrasound imaging unveiled a well-defined, approximately 11×8 mm hypoechoic nodule in the left breast. The lesion exhibited irregular morphology with an aspect ratio greater than 1. Echoes remained homogeneous, and no noticeable blood flow signals were observed. **(B)** SWE imaging displayed a relatively soft nodular texture, with an Emax value of 45 kPa. **(C)** Following a bolus injection of 4.8 mL contrast agent through the superficial vein at the elbow, enhancement was monitored at 7 seconds, preceding the enhancement in surrounding glandular tissues. Peak enhancement was attained at 14 seconds, demonstrating slightly lower-level and homogeneous enhancement. The lesion exhibited a clear boundary and a surrounding ring-like enhancement pattern. Post-enhancement, the lesion’s size remained unchanged, and washout-type alterations occurred earlier than in the surrounding glandular tissues. **(D)** Pathological examination yielded a diagnosis of fibroadenoma of the breast.

**Figure 2:**
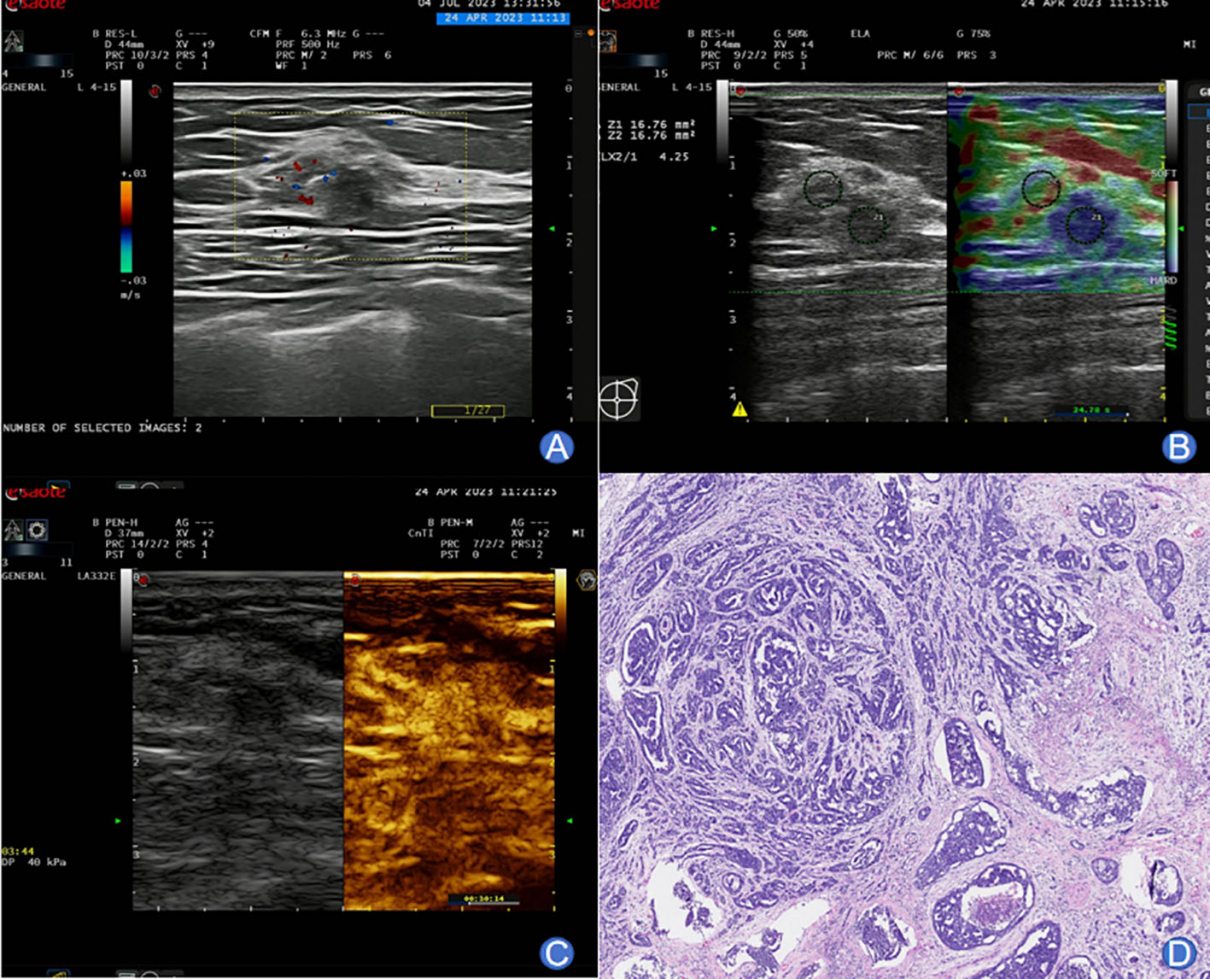
A 59-year-old female presenting typical images of malignant breast tumor. **(A)** Conventional ultrasound imaging revealed an irregularly shaped, partially indistinct, approximately 8×6.7 mm lesion within the right breast. The internal echoes were heterogeneous, and the surrounding tissue structure appeared distorted. Peripherally, blood flow signals were detected. **(B)** SWE imaging displayed a relatively stiff nodular texture, with an Emax value of 97 kPa. **(C)** Following a bolus injection of 4.8 mL SonoVue contrast agent, enhancement was observed at 13 seconds, reaching the peak at 28 seconds. The lesion exhibited centripetal high enhancement with the presence of large blood vessels entering. Enhancement extended beyond the range of 2D imaging, with an irregular morphology and unclear boundary. The washout occurred later than in the surrounding glandular tissues. **(D)** Pathological examination confirmed invasive ductal carcinoma of the non-special type.

The typical images and pathological outcomes of benign and malignant breast tumors are exhibited in **Figures 1**, **2**.

### 3.5 ROC curve analysis of S-CEUS quantitative analysis, SWE alone, or combined assessment for breast tumor characterization

**Figure 3:**
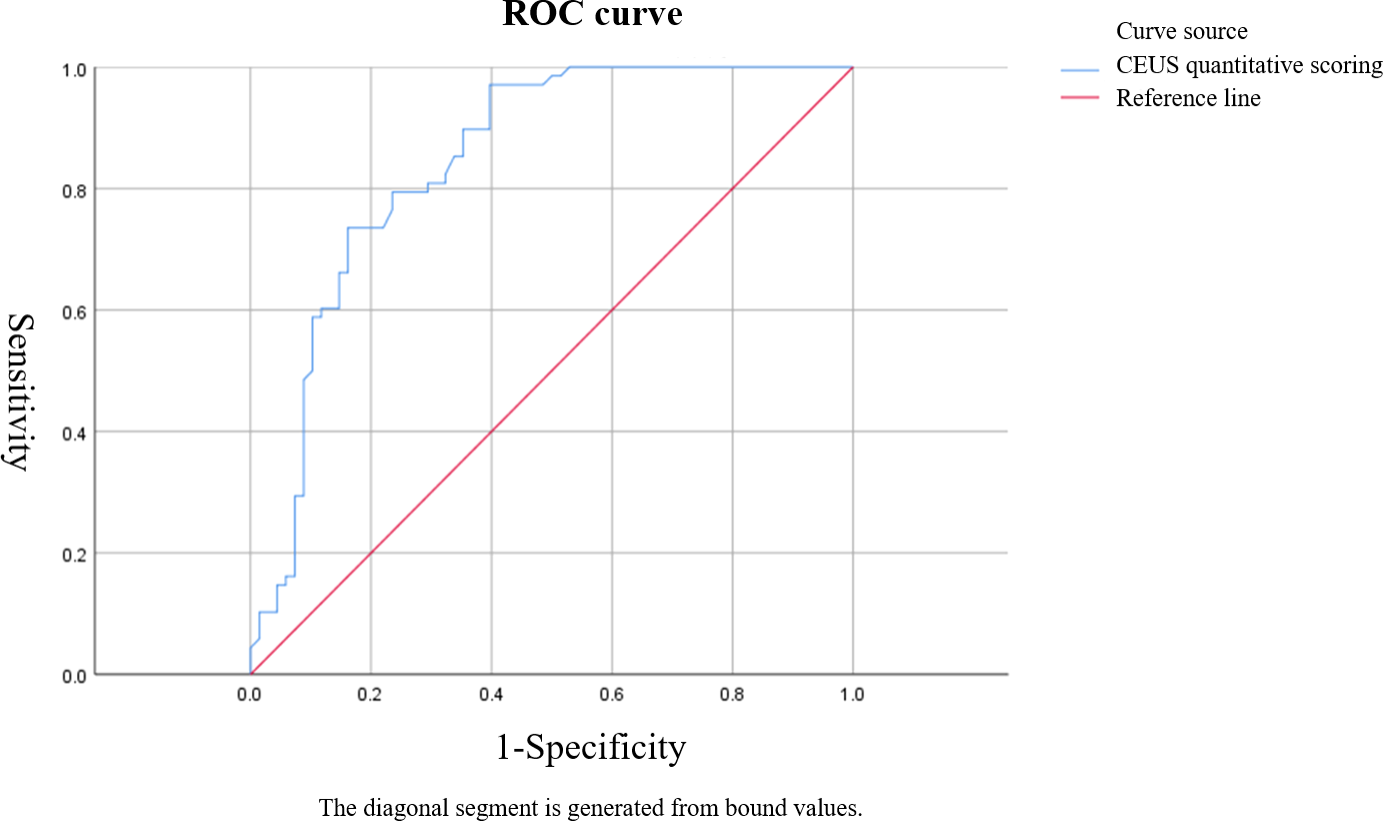
ROC curve of Sonazoid-contrast-enhanced ultrasound (S-CEUS) quantitative analysis in characterizing breast tumors. The area under the curve (AUC) was 0.845, with an optimal cutoff score of 3.40, yielding a sensitivity of 73.53% and specificity of 84.61%.

**Figure 4:**
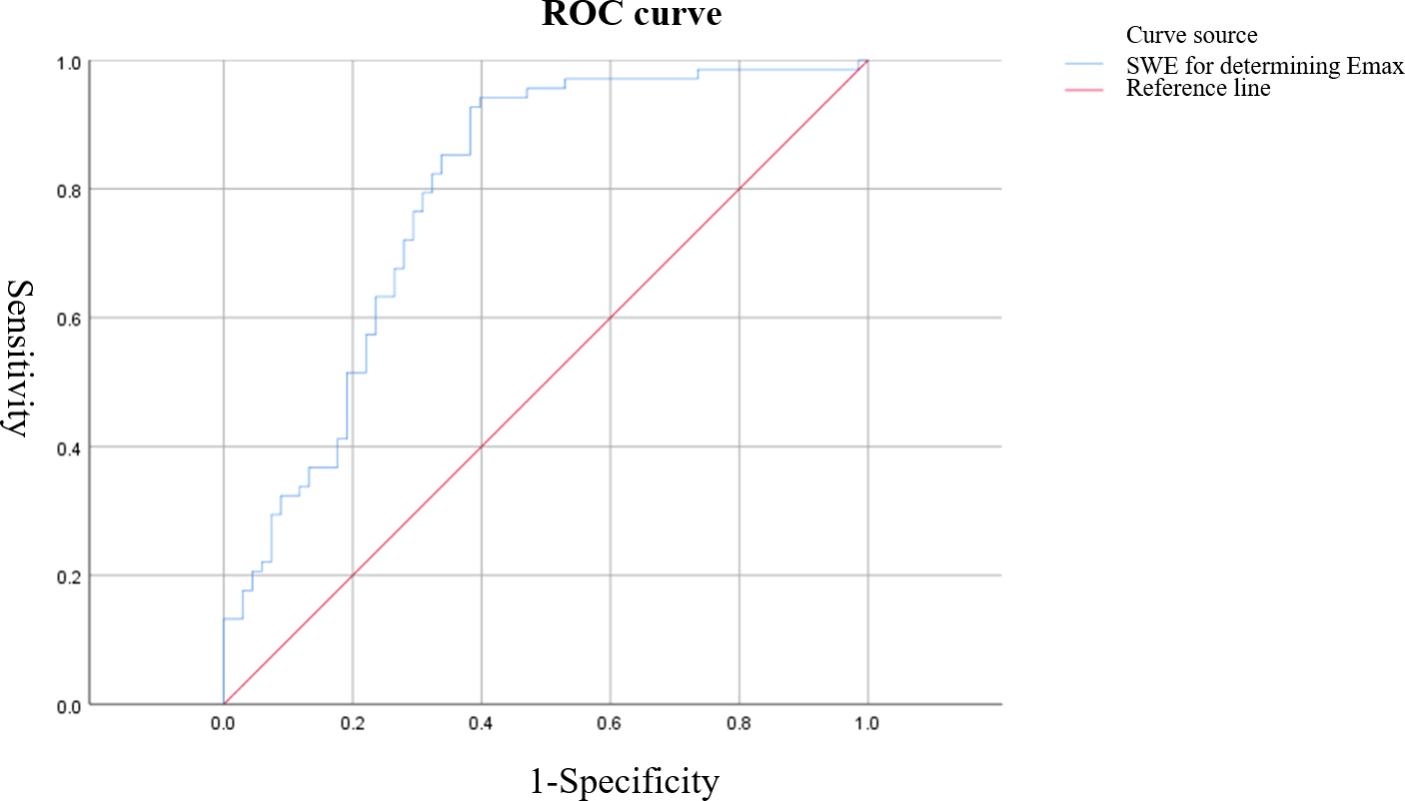
ROC curve of shear wave elastography (SWE) Emax value for characterizing breast tumors. The AUC was 0.789, with an optimal cutoff value of 57.38 kPa, resulting in a sensitivity of 92.60% and specificity of 61.54%.

**Figure 5:**
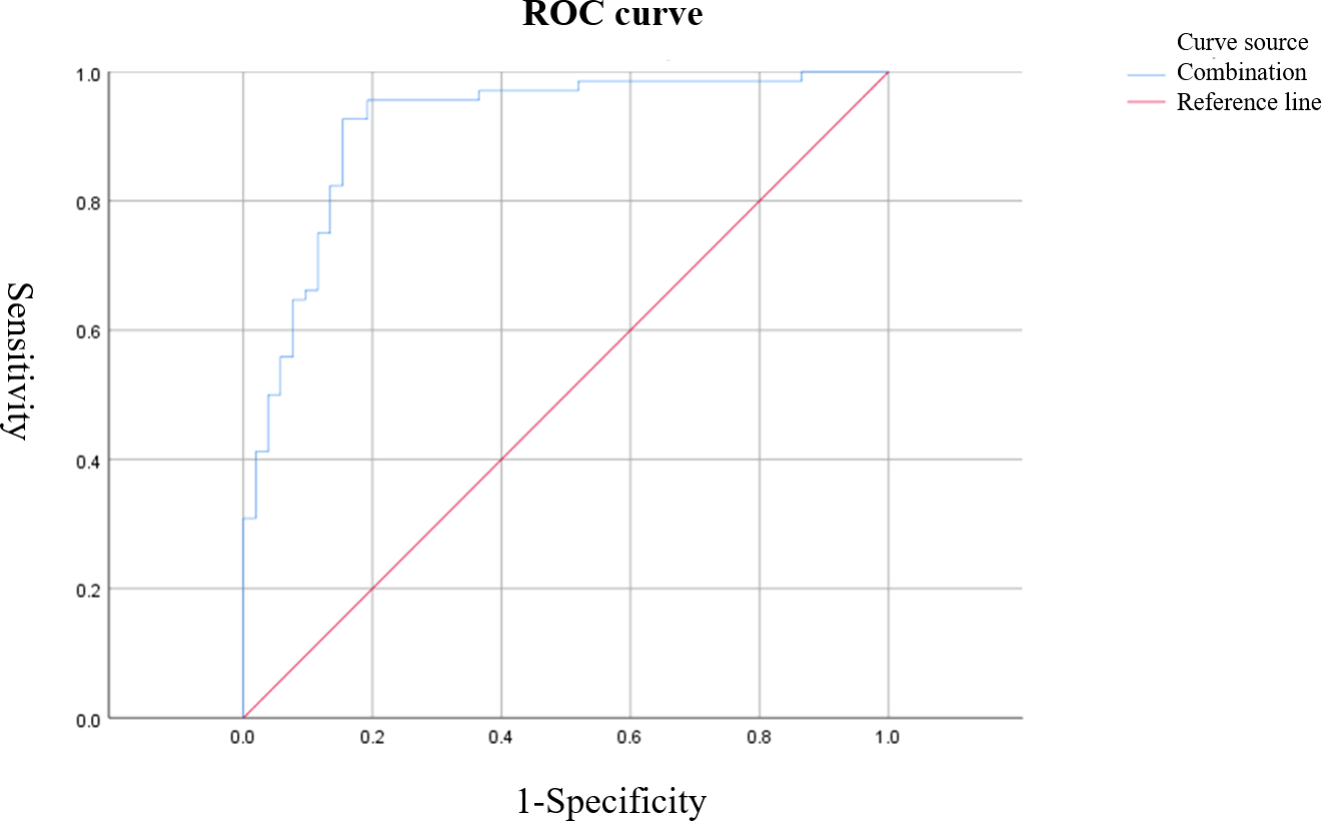
ROC curve of combined S-CEUS quantitative analysis and SWE for characterizing breast tumors. The combined diagnostic approach achieved the highest performance with an AUC of 0.916, sensitivity of 95.59%, and specificity of 80.77%.

Based on ROC curve analysis, the diagnostic performance for characterizing breast tumor nature was relatively favorable for S-CEUS quantitative analysis or SWE alone, with corresponding area under the curve (AUC) values of 0.845 and 0.789, respectively. Particularly noteworthy was the enhanced diagnostic efficacy achieved through the combination of S-CEUS quantitative analysis and SWE, yielding an optimal AUC value of 0.916. At this juncture, the sensitivity and specificity were recorded as 95.59% and 80.77%, respectively. Refer to **Table 2** and **Figures 3**-**5** for detailed outcomes.

**Table 2:** ROC curve analysis of S-CEUS quantitative analysis, SWE alone, or combined assessment for breast tumor characterization.

Inspection method	ROC curve			Optimal cutoff value	Sensitivity (%)	Specificity (%)
	AUC value	95% CI value	p value			
S-CEUS quantitative analysis	0.845	0.777~0.913	0.000	3.40 points	73.53 (50/68)	84.61 (44/52)
SWE for determining Emax	0.789	0.711~0.867	0.000	57.38kPa	92.60 (63/68)	61.54 (32/52)
Combined	0.916	0.863~0.969	0.000	/	95.59 (65/68)	80.77 (42/52)

## 4 Discussion

Due to the relatively high incidence and mortality rates of malignant breast tumors, they can significantly impact the quality of life of patients. Therefore, the early and accurate determination of the benign or malignant nature of breast tumors holds crucial significance. Pathological biopsy serves as the gold standard for distinguishing the nature of breast tumors; however, its invasive nature limits its applicability (16). Consequently, routine ultrasound examination is frequently employed in clinical practice, often relying on the BI-RADS classification system to categorize breast tumors. Nevertheless, the malignant risk threshold for BI-RADS category 4 breast lesions is relatively broad. This is attributed to the diverse pathological features of breast tumor diseases, coupled with the variability in ultrasound appearances. As a result, there can be overlapping and intersecting characteristics when determining the benign or malignant nature of breast tumors (17). For instance, the ultrasonic images of lobular inflammatory granuloma, a benign breast tumor, may resemble those of breast cancer, both predominantly exhibiting low echoes, unclear boundaries, and irregular morphology (18). Consequently, relying solely on conventional ultrasound and BI-RADS classification may not suffice for definitively determining the nature of breast tumors.

Due to the production of abundant vascular endothelial growth factor (VEGF) by malignant breast tumors, excessive neovascularization occurs. Furthermore, in order to sustain the growth of tumor cells (primarily manifested as infiltrative growth), the vasculature within the tumor exhibits a predominantly tortuous and disordered pattern. Additionally, these proliferative vessels display reduced luminal diameter, thin vessel walls, and deficient smooth muscle cells, resulting in incomplete endothelial structure. Consequently, compromised vasoconstriction and diastolic functions lead to high-level blood perfusion. Thus, significant disparities exist in the microcirculation of patients with malignant breast tumors compared to those with benign tumors (19, 20). A novel pure blood pool imaging technique based on ultrasound known as CEUS presents a valuable approach to address the limitations of conventional ultrasound. By administering a Sonazoid contrast agent via the elbow vein, CEUS enables the visualization of microvascular structures within breast masses, facilitating the acquisition of information pertaining to morphological characteristics and arterial enhancement features of these lesions (21). The findings of this research demonstrated that, in comparison to benign breast tumors, malignant tumors showcased irregular morphologies, indistinct boundaries, heterogeneous internal enhancements, elevated enhancement levels, expanded enhancement ranges, presence of perfusion defects, and predominant washout-type TIC. Consequently, this study summarized the key CEUS features of benign and malignant breast tumors. The former is primarily characterized by regular morphology (predominantly circular or oval), clear boundaries, and low or iso-enhancement. In contrast, malignant tumors commonly present with irregular shapes, ill-defined boundaries, “crab claw” alterations in morphology, heterogeneous high enhancement, perfusion defects, and an increased post-enhancement lesion area. These discrepancies stem from the rapid metabolic growth of malignant breast tumors, resulting in increased tumor volume. Uneven distribution of newly formed blood vessels and reduced microvascular density at the center of the tumor contribute to inadequate blood supply, leading to fibrotic necrosis and subsequent perfusion defects evident during CEUS. Moreover, manifestations also encompass heterogeneous enhancement and the higher microvascular density at the tumor periphery than in the central region, leading to elevated enhancement levels and potential ring enhancement. Consequently, lesions are enlarged subsequent to CEUS enhancement, potentially accounting for the limitations of conventional ultrasound in depicting alterations in the surrounding tissue of the tumor (22). The Chinese preliminary 5-point CEUS scoring system is generally based on the aforementioned indicators and serves as a practical quantitative criterion for breast tumor diseases. Furthermore, a prospective study by Xiao et al. (23) demonstrated that the 5-point scoring system for differentiating benign and malignant breast tumors yielded an AUC value of 0.895, with sensitivity and specificity of 82.10% and 96.90%, respectively. The findings of this research reflected that S-CEUS quantitative analysis provided a favorable diagnostic efficacy of breast tumor nature, with an AUC value of 0.845 and sensitivity and specificity of 73.53% and 84.61%, respectively. While in concurrence with the aforementioned research, the diagnostic performance was slightly lower, potentially attributed to variations in participant heterogeneity, operator proficiency, and contrast agent selection. Additionally, given the above findings, it is noteworthy that even with the implementation of S-CEUS quantitative analysis, the possibility of missed diagnoses and misdiagnoses remains. This is due to the fact that inflammatory granulomas also feature abundant neovascularization, and the proliferative infiltration of inflammatory cells into peripheral tissues can result in characteristics such as unclear boundaries, heightened enhancement, and expanded range, leading to possible confusion with features of malignant breast tumors and subsequent misdiagnosis (24, 25). On the other hand, in early-stage invasive ductal carcinoma, the relative scarcity of fibrous components and limited blood supply might not yet manifest infiltration or necrosis, thus presenting benign-like features that can give rise to missed diagnoses (26). Internal enhancement characterized by non-enhancement or homogeneous enhancement is more commonly observed in benign masses, whereas heterogeneous enhancement is prevalent in malignant tumors. However, it should be noted that invasive breast cancer with a diameter less than 10 mm can also exhibit signs of homogeneous enhancement. As such, the current research focus lies in combining S-CEUS quantitative analysis with other inspection methods. Our results demonstrated that while S-CEUS and SWE each had moderate diagnostic efficacy when used alone (AUCs of 0.845 and 0.789, respectively), the combination significantly improved accuracy (AUC 0.916), with enhanced sensitivity (95.59%) and specificity (80.77%). These findings confirm the complementary nature of these modalities and support their integrated application in clinical practice.

After the occurrence of malignant breast tumors, alterations in the tissue’s biological characteristics within the tumor mass transpire (specifically, the infiltrative growth of tumor cells is rapid; in order to sustain the processes of division and proliferation, adequate supply of nutrients should be ensured; in instances of insufficient nutrient provision, tumor cell necrosis may ensue, subsequently inducing the proliferation of adjacent normal tissues into fibroblasts, achieving fibrous tissue repair through chemotactic aggregation), ultimately culminating in diminished tissue elasticity and heightened hardness (27). SWE, on the other hand, involves the emission of acoustic radiation pulse waves into tissues via a probe. Under indirect external action, tissue formation occurs, causing continuous aggregations within the tissue at varying depths and thus forming a phenomenon akin to a “Mach cone”. This phenomenon engenders shear waves, whose propagation speed within the tissue can be quantitatively measured to ascertain the tissue’s elasticity modulus. Studies by Farooq et al. (28) have demonstrated that an SWE-measured Emax cutoff value of 72 kPa yields sensitivity and specificity values of 92.17% and 90.4%, respectively. Our study unraveled that the standalone SWE assessment of breast tumor nature exhibited favorable diagnostic efficacy, with an AUC value of 0.789, along with sensitivity and specificity values of 92.60% and 61.54%, respectively. While the diagnostic efficacy slightly lags behind that reported in foreign studies, this disparity is potentially attributed to ethnic differences and variances in ROI selection. These observations align with domestic research findings (29), illustrating that relying solely on SWE for discerning benign and malignant breast tumors may result in the potential for missed diagnoses and misdiagnoses. This is caused by various factors, including: 1) The intrinsic characteristics of tumors: When tumors are located at greater depths or display uneven surfaces, acquired SWE images can easily become distorted; 2) Varied pathological changes in different types of breast tumors: For instance, mucinous carcinoma is primarily composed of mucin, rendering it relatively less rigid; 3) Differences in ROI selection: Presently, two predominant ROI selection strategies are employed within China. The first aims to encompass the tumor while avoiding the surrounding area of increased hardness and also seeks to exclude normal tissues. The second involves placing an ROI at the hardest point within or surrounding the tumor. However, the precise clinical utility of each approach remains ambiguous.

To conclude, it is evident that both S-CEUS quantitative analysis and SWE have inherent limitations when distinguishing benign from malignant breast tumors. Nevertheless, the higher sensitivity exhibited by SWE-derived Emax values can potentially compensate for the shortcomings of S-CEUS quantitative analysis. Our findings corroborated that the optimal diagnostic efficacy was achieved through the combined use of S-CEUS quantitative analysis and SWE, resulting in an AUC value of 0.916, with sensitivity and specificity values being 95.59% and 80.77%, respectively, suggesting the practical viability of utilizing S-CEUS quantitative analysis in conjunction with SWE for evaluating the nature of breast tumors. Additionally, Xiang et al. (30) have shown that CEUS combined with SWE can aid in distinguishing benign from malignant breast tumors with diameters smaller than 10 mm, further substantiating the accuracy of our study’s discoveries. Nevertheless, this research displays the following limitations: 1) Due to its relatively small sample size and lack of representativeness, the study relies on previously established, relatively reliable CEUS quantitative analysis and SWE techniques, without the formulation of a more robust and precise quantification standard; 2) The study is a single-center retrospective investigation, thereby introducing certain biases into the research conclusions.

In summary, S-CEUS quantitative analysis and SWE can both be employed for the assessment of the benign-malignant nature of breast tumors, with particular emphasis on the optimal diagnostic performance achieved through their combined application.

## Data Availability

The raw data supporting the conclusions of this article will be made available by the authors, without undue reservation.
